# The Effects of Perceived Mating Opportunities on Patterns of Reproductive Investment by Male Guppies

**DOI:** 10.1371/journal.pone.0093780

**Published:** 2014-04-04

**Authors:** Luke T. Barrett, Jonathan P. Evans, Clelia Gasparini

**Affiliations:** Centre for Evolutionary Biology, School of Animal Biology, The University of Western Australia, Crawley, Western Australia, Australia; National Cancer Institute, United States of America

## Abstract

Males pay considerable reproductive costs in acquiring mates (precopulatory sexual selection) and in producing ejaculates that are effective at fertilising eggs in the presence of competing ejaculates (postcopulatory sexual selection). Given these costs, males must balance their reproductive investment in a given mating to optimise their future reproductive potential. Males are therefore expected to invest in reproduction prudently according to the likelihood of obtaining future matings. In this study we tested this prediction by determining whether male reproductive investment varies with expected future mating opportunities, which were experimentally manipulated by visually exposing male guppies (*Poecilia reticulata*) to high or low numbers of females in the absence of competing males. Our experiment did not reveal consistent effects of perceived future mating opportunity on either precopulatory (male mate choice and mating behaviour) or postcopulatory (sperm quality and quantity) investment. However, we did find that male size and female availability interacted to influence mating behaviour; large males visually deprived of females during the treatment phase became more choosy and showed greater interest in their preferred female than those given continuous visual access to females. Overall, our results suggest males tailor pre- rather than postcopulatory traits according to local female availability, but critically, these effects depend on male size.

## Introduction

In order to reproduce successfully, males often have to balance their investment in traits that enhance their access to mates (e.g. courtship, weapons) and those that increase the likelihood that their sperm will compete effectively for fertilisations (e.g. ejaculate size or quality). Because both mating acquisition and sperm production are costly, and the energy available to organisms is limited, the investment in a given mating limits the opportunities and the energy available for future reproduction (e.g. [1], [2]), generating a trade-off between current and future reproductive investment [3]. In light of such constraints, theory predicts that, where mating is costly, the number of available females is high and there is variation in the reproductive return of a mating, males should strategically allocate their current mating effort according to both female quality and the opportunity for future matings, leading to the evolution of pre- and postcopulatory male choice [4]–[9].

Despite the expectation that males should strategically allocate their reproductive effort (both pre- and postcopulatory) according to mate availability (theory reviewed by [6], [8]–[10]), empirical evidence to support this prediction is limited to just a handful of studies, particularly in the context of postcopulatory reproductive investment. In the fowl (*Gallus gallus domesticus*), for example, dominant males expend fewer sperm per mating according to the availability of females, thus preserving energy for future matings [11]. Indirect evidence for adaptive changes in resource allocation according to perceived future mating opportunities comes from scorpionflies (*Panorpa cognata*), where males become less discriminating as they age, probably due to their lower expected future matings that decline with age [12]. Similarly, a male’s own attractiveness may influence his patterns of reproductive investment in species where female choice occurs. According to this idea, more attractive males will have relatively higher future mating opportunities compared to less attractive males and therefore will be more likely to exhibit mate choice [8]. Despite ongoing progress in demonstrating adaptive variation in patterns of male reproductive investment, no study has considered investment in both the pre- and postcopulatory episodes of sexual selection when testing for responses to expected future mating opportunities.

In this study, we determine whether experimental manipulation of perceived future mating opportunities influences reproductive investment by male guppies (*Poecilia reticulata*). Guppies are a sexually dimorphic, polyandrous internally fertilising poeciliid fish subject to intense pre- and postcopulatory sexual selection [13], [14]. Males may achieve matings through both courtship and forced (sneaky) copulations. Courtship is known to be costly for males [15]–[19], while sperm limitation is implied by the presence of a postcopulatory refractory period and the several days required to replenish sperm reserves [20]–[22]. Some degree of male choosiness and adaptive plasticity in reproductive investment is therefore expected. Indeed, prior work has shown that male guppies exhibit mate choice [23]–[28], and that the strategy of male choice is influenced by male’s own relative attractiveness to females [28]. Furthermore, there is evidence for plasticity in courtship effort [29], sperm production [30] and sperm quality(sperm swimming velocity: [31]), all of which predict male reproductive success in guppies [32], [33]. A recent study also found that male guppies use past experience to adjust mating behaviour, indicating that males are able to modify their mate preference to maximize their fitness in changing social environments [34].

To determine whether males adjust their reproductive investment in pre- and postcopulatory traits according to perceived future mating opportunities, we visually exposed males to high or low numbers of females in the absence of competing males (to remove the direct influence of sperm competition), and simultaneously assessed responses in pre- and postcopulatory investment. We expected that males exposed to high numbers of available females would (a) exhibit heightened choosiness during mate choice experiments, and (b) direct less pre- and postcopulatory effort towards a single mating opportunity in order to conserve energy and resources for subsequent matings. By incorporating traits in our analyses associated with male attractiveness, such as body size and colouration, we were also able to determine whether, and to what extent, male attractiveness interacts with female availability in shaping mate strategies and reproductive allocation.

## Materials and Methods

### Ethics Statement

The measures of precopulatory traits did not involve any invasive manipulations, and were performed in conditions that mimic natural conditions. For the postcopulatory trait analysis, fish were anaesthetised to render them immotile during procedures (e.g. sperm extraction, photograph) through immersion in a water bath containing MS222 (tricaine methanesulfonate) before handling or sperm collection. Sperm extraction from males and sperm collection from the females’ reproductive tract, are widely used techniques in guppies, with no impairment of the individuals’ health (e.g. [35]). This study was carried out in accordance with the Australian Code of Practice for the Care and Use of Animals for Scientific Purposes. The work was approved by the University of Western Australia Animal Ethics Committee (permit number: RA/3/100/1050).

### Experimental Overview and Housing of Experimental Subjects

The guppies used in this study were laboratory-reared descendants of fish obtained in 2006 (which corresponds to approximately 18 guppy generations) from Alligator Creek, Queensland (the original collection permit was issued by the Environmental Protection Agency, Queensland Parks and Wildlife Service). Guppies were housed in numerous mixed-sex aquaria (115 L tanks) until used in this study (with regular rotation of fish to minimise inbreeding). All experiments were conducted in temperature- and photoperiod-controlled rooms over a four month period in 2012–2013. Adult male guppies were haphazardly taken from our stock population and allocated at random to treatments simulating two levels of female availability: high female availability (HFA, four females in view) and low female availability (LFA, no females in view). In both treatments, males were housed with a female twice a week for four hours to maintain sexual activity (see treatment regime below). After 35 days, trials were conducted to compare pre- and postcopulatory investment between treatment groups. Male mate choice and sexual interest was assessed using a dichotomous mate choice trial, in which each focal male was given a simultaneous choice between two females that differed in body length (with expectation that males should prefer larger females – see below). Males were then moved to a tank containing a virgin female, where mating effort was quantified by the number of sigmoid displays (courtship) and gonopodial thrust attempts (forced copulations) (see *Precopulatory Traits* below for description of these behaviours). Following these trials, copulation success was assessed based on the presence/absence of sperm in the female gonoduct, while males were stripped of remaining sperm to assess sperm quality (viability, velocity and length) and sperm counts (i.e. total sperm remaining after the mating trials).

### Treatment Regime

Eighty focal males (*n = *40 per treatment,) underwent their respective treatments (HFA & LFA) for 35 days, spanning an entire spermatogenesis cycle [36]. Males were individually housed in plastic tanks (19×11×11 cm, filled to 9 cm) containing gravel, while an identical tank containing either four (HFA) or zero (LFA) females was placed adjacent to each male’s tank. The HFA level was chosen to approximate the upper bounds of mate availability in wild populations [37]. The tanks housing the females were moved between focal males in the HFA treatment on a weekly basis, as males typically show reduced interest in familiar females [23]. A randomly chosen non-virgin female (termed a companion female) was introduced into the male tanks (for both treatment groups) for four hours twice weekly to maintain normal sexual activity during the treatment phase [13], but withheld for the final three days of treatment to ensure sperm replenishment by the focal males [21].

### Precopulatory Traits

Initial behavioural trials were designed to evaluate sexual interest and compare the strength of male choosiness between treatments. To achieve this, following the treatment phase (i.e. on day 36) each focal male was visually presented with two females of differing size in a dichotomous choice chamber, with the expectation that males assigned to HFA treatment would exhibit heightened choosiness in favour of preferred (large) females compared to their LFA counterparts. Male guppies have previously been shown to prefer larger females [24]–[26], [38], [39], presumably due to their higher fecundity. We chose to use a dichotomous choice test in order to present males with a simultaneous choice of females, a condition that mimics the common mating system of guppies. The choice apparatus consisted of two adjacent tanks (35×19×22 cm, filled to 13 cm), with one for the focal male and the other for the two stimulus females. The female tank was divided into two identical sectors with an opaque barrier, with a large female on one side and small female on the other. Females were randomly chosen from pools of 40 large (standard length 26–37 mm) and 40 small (standard length 17–30 mm) females and settled in the trial tank for one hour prior to the trial. (Note that although there was slight overlap in the size of large and small females, we selected females by eye for each trial to ensure that the pair always differed in size by at least 5 mm.) The relative position (left or right) of the stimulus females was randomised in successive trials. Each male was allowed a 30 minute acclimation period prior to the mate choice trial, with a screen preventing any visual contact among the fish during this time. The screen was then gently removed and the male allowed to observe the stimulus females for 20 minutes. To ensure that the male viewed both females concurrently from a neutral position, the male was initially positioned within a transparent plastic cylinder (12 cm diameter) in the centre of his tank (i.e. equidistant from the female sectors).

We recorded the time spent by the male in front of each female sector (within 10 mm of the glass and facing the female) over a 20 minute period. Variations on this trial format have been used widely in poeciliids (e.g. [25], [28]), and it has been shown to accurately reflect male mating preference in guppies [40]. An index of choosiness was obtained by dividing the difference between the time spent in front of the preferred female by the total time spent in front of both females, giving a proportional choosiness index between zero (equal time spent with both females) and one (entire time spent with the preferred female). We also obtained estimates of male ‘sexual interest’, which came from measures of the combined time spent in front of both stimulus females as a proportion of the trial duration.

Immediately following each choice trial, the male was moved to a tank containing a virgin female (standard length: 19–25 mm; age: ca. 4 months) that had been settled for two hours. Virgin females were used for the mating trials because they exhibit heightened sexual activity and receptiveness compared to non-virgin females [13]. The number of courtship displays (sigmoids) and forced copulation attempts (gonopodial thrusts) were recorded during the first 20 minutes of the trial. Sigmoids were recorded when the male positioned himself in front of the female, assumed a lateral s-shaped posture, and quivered, while gonopodial thrusts were recorded when the male approached the female from behind, swung his gonopodium forward ≥90° from its resting position, and attempted to inseminate the female without prior display [20]. After the observation period, the pair was left for a further 100 minutes (120 minutes in total) to increase the likelihood of copulation. Copulation success was determined based on the presence of sperm in the female reproductive tract following this trial (see *Postcopulatory Traits* below for sperm collection procedures).

### Postcopulatory Traits

After the mating trial, both the male and the female were anaesthetised for sperm extraction. Sperm were retrieved from the anaesthetised female by flushing the genital tract with 0.9% NaCl physiological solution [41], while sperm were collected from males by applying light pressure to the male’s abdomen to release sperm into an inactivating (extender) solution where sperm remain quiescent [42]. From the latter samples, six bundles were used for sperm velocity assays, and ten for sperm viability assays; the remaining bundles were gently broken up into free sperm using a pipette and vortex to enable sperm count. Each male’s sperm count (excluding the sperm taken for velocity and viability assays) was estimated using an improved Neubauer haemocytometer.

The proportion of viable sperm was measured using a fluorescent live/dead staining kit (L-7011, Molecular Probes, Inc., Eugene, OR, USA). Sperm were stained using an established protocol (see [43]), the solution was transferred to a slide, and a count of live and dead spermatozoa was made under UV light. A minimum of 100 spermatozoa were scored from each male.

Sperm swimming velocity was measured using computer-assisted sperm analysis (CASA; CEROS software: Hamilton Thorne, Beverly, MA), using an established protocol designed for guppies [31]. Briefly, a 3 μl aliquot of activating solution (150 mM KCl) was placed into each of two duplicate slide wells (pre-treated with 1% polyvinyl alcohol solution to prevent sperm sticking), and three bundles were placed into each well along with 2 μl of the extender medium. The velocity of freely swimming spermatozoa leaving the bundles was tracked using the CASA software (tracks/sample ± SD = 144.6±94.9) and included: average path velocity (VAP, μm s^−1^), curvilinear velocity (VCL, μm s^−1^), and straight line velocity (VSL, μm s^−1^). As in previous studies, VAP was highly correlated with both VSL and VCL (both Pearson r-values >0.78, *P*<0.001), and therefore we present results for VAP only (results were similar for VCL and VSL). For each ejaculate, two samples were analysed, and given significant repeatability [44] within males (*R* SE = 0.608±0.067, *N* = 63, *P*<0.0001) the mean of the two measures was used.

Photographs of 20 spermatozoa from each male were taken at 40× objective magnification using a digital camera on a light microscope (Leica DM1000). Spermatozoa were chosen haphazardly, provided they were intact and visible along their entire length. We then used ImageJ (http://rsbweb.nih.gov/ij) software to estimate the length of each sperm cell’s head, midpiece and flagellum. All sperm assays were performed blind of the experimental treatment to eliminate observer bias.

### Male Body Size and Colouration Measurements

After sperm extraction, each male was photographed (left side) using a digital camera. Male body size (standard length = distance in millimetres from the snout to the tip of caudal peduncle) and the area of orange and iridescent (combined white, purple, green and blue) spots was measured using ImageJ software. The relative area of coloured spots on body area was used in analyses after arcsin square-root transformation.

### Statistical Analyses

All analyses were performed using R software version 2.15.3 [45]. We test for an effect of treatment (fixed effect) on a number of response variables, including precopulatory ‘traits’ (choosiness, sexual interest, sigmoid count, gonopodial thrust count and copulation success) and postcopulatory traits (sperm number, proportion viable sperm, sperm velocity, and sperm head, midpiece and flagellum length). Response variables were transformed where necessary to approximate normality and homogeneity of variances. Error distributions were not consistent among response variables; precopulatory traits and sperm count, were modelled using univariate models with appropriate error distributions for each trait, while all sperm quality traits exhibited similar error distributions and were therefore considered together in a general linear multivariate model. Among the precopulatory traits, choosiness and sexual interest were modelled by general linear models, while copulation success was modelled by a binomial generalised linear model. Data for sigmoid display and gonopodial thrust counts were overdispersed and were therefore modelled by quasi-Poisson generalised linear models. As males did not show a significant preference for the larger female in either treatment (one sample t-tests: LFA *t*_36_ = 0.409, *P* = 0.685; HFA *t*_39_ = 1.475, *P* = 0.148), we used the index of choosiness in which time spent with the ‘preferred’ female was considered regardless of her size category (providing a measure of choosiness that is independent of female size).

Male body size, orange area and iridescent area were fitted as covariates to all full models, and female size was fitted as a covariate when constructing models for sigmoid, gonopodial thrust and copulation success response variables. When analysing sperm number, copulation success was included as a factor to account for sperm depletion in males that had mated before sperm extraction. There was no significant treatment-covariate collinearity or heterogeneity of variances across treatments, and the only significant association between male morphological covariates involved iridescence and male size (Pearson: *t*_75_ = 2.34, *P* = 0.022, *R* = 0.26). Both covariates were included in the model selection process, which was conducted by specifying all appropriate covariates and interactions in a full model, then searching for the best model containing the treatment main effect using the MuMIn package [46]. Candidate models were ranked by second-order Akaike’s Information Criterion (AIC_c_ or quasi-AIC_c_) [47], with only the best model interpreted. Type II sums of squares were calculated for general linear models and multivariate significance was tested using Pillai’s trace statistic (R package car, [48]).

Where models revealed a treatment-by-covariate interaction, following Engqvist [49] we performed a modified Johnson-Neyman procedure by using the R package jnt [50] for establishing regions of non-significance throughout the range of the covariate (in our case male body size). Note that the lower confidence interval is not presented in the figure as it is outside the male size range examined.

Three LFA males that appeared unhealthy during the course of treatment phase were removed from the experiment, thus the final sample size was N = 77. For some males it was not possible to measure all the sperm traits, and so the final sample sizes relative to each trait is reported in Table 1.

**Table 1:** Descriptive statistics of male traits in the two experimental groups.

	LFA males		HFA males	
	(Mean ± SD)	(N)	(Mean ± SD)	(N)
**Precopulatory traits:**				
*Choosiness*	0.55±0.29	37	0.49±0.30	40
*Sexual interest*	0.78±0.17	37	0.71±0.18	40
*Sigmoids*	11.59±25.18	37	9.07±18.47	40
*Gonopodial thrusts*	10.78±13.99	37	17.85±25.22	40
*Copulation success rate*	0.29±0.46	37	0.20±0.40	40
**Postcopulatory traits:**				
*Sperm velocity (μm/s)*	76.2±13.03	34	74.11±16.55	38
*Sperm viability*	0.84±0.12	33	0.83±0.19	39
*Sperm count (x 10^6^)*	2.96±2.59	34	2.67±1.78	39
*Sperm head (μm)*	3.9±0.10	31	3.88±0.07	38
*Sperm midpiece (μm)*	5.23±0.98	31	5.27±1.05	38
*Sperm flagellum (μm)*	45.7±1.34	31	45.45±1.2	37

Mean, standard deviation (SD) and sample size (N) are reported for the low (LFA) and high (HFA) female availability groups. Choosiness and sexual interest are indexes; sigmoid displays and gonopodial thrusts are reported per hour; sperm viability is expressed as a proportion. See main text for detailed description of each trait.

## Results

### Precopulatory Traits

**Figure 1:**
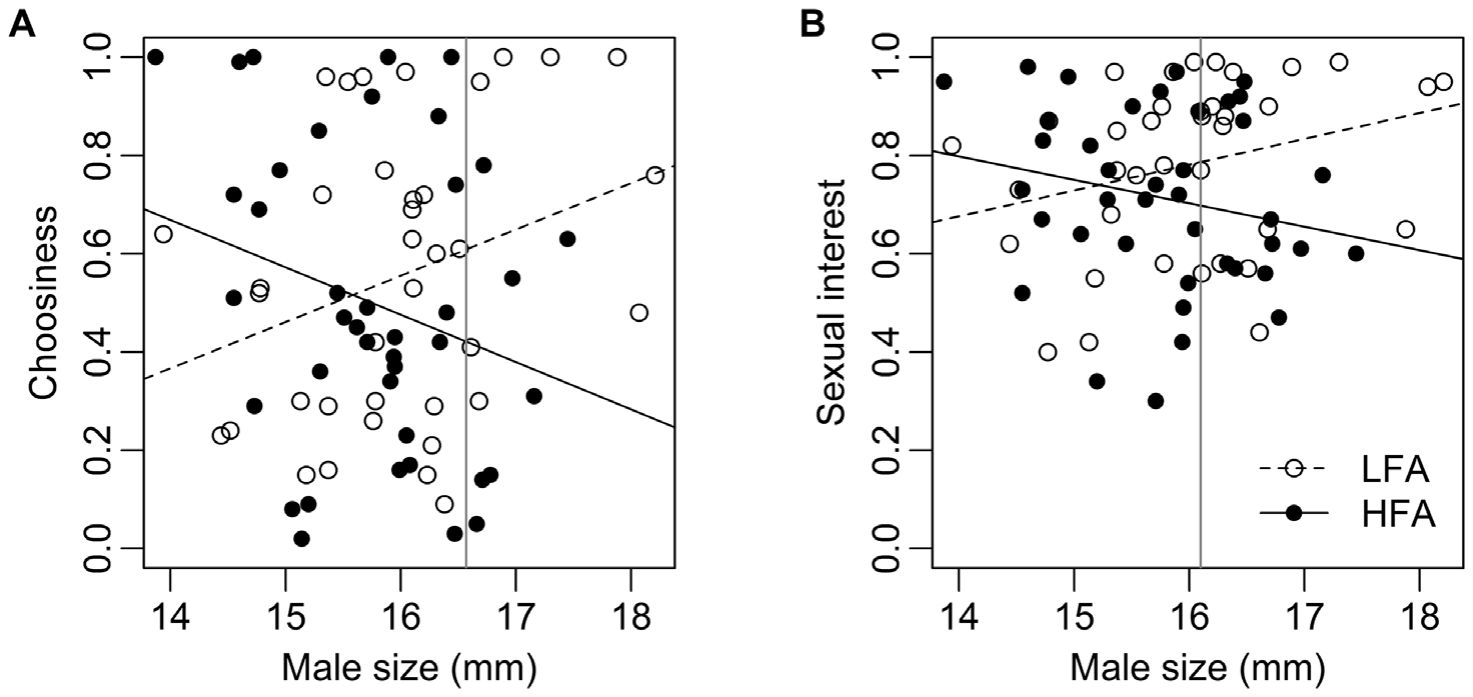
Interactions between female availability and male size on precopulatory sexual behaviour in guppies: (a) choosiness, (b) sexual interest (see text for details). Low (LFA) and high (HFA) female availability treatment groups are plotted separately. The vertical line (a: 16.6 mm, b: 16.1 mm on X axis) represents the upper limit of the Johnson-Neyman nonsignificance 95% confidence interval.

There was no consistent treatment effect on male sexual choosiness. Instead, our analysis revealed a significant effect of iridescence, with more coloured males being more choosy than less ornamented males. Furthermore, we detected a significant interaction between treatment and male body size on male sexual choosiness (Table 2a). The Johnson-Neyman procedure indicated that only large males significantly responded to female availability, with LFA males exhibiting heightened choosiness over their HFA counterparts outside the region of non-significance of the covariate (body size) (Fig. 1a).

**Table 2:** Effect of female availability treatment on precopulatory traits in the guppy.

Source	*df*	Estimate	SS	Statistic	*P*	Effect size
***(a) Choosiness***				*F*		
Treatment	1	0.057 (–0.109, 0.223)	0.0557	0.426	0.516	0.16 (–0.30, 0.62)
Male size	1	–0.151 (–0.293, –0.010)	0.0179	0.137	0.713	–0.08 (–0.55, 0.38)
Iridescence	1	1.440 (0.221, 2.659)	0.7255	5.542	**0.021**	0.56 (0.09, 1.02)
Treatment×Male size	1	0.245 (0.055, 0.434)	0.8672	6.624	**0.012**	0.61 (0.14, 1.08)
Residuals	72		9.4255			
***(b) Sexual interest***				*F*		
Treatment	1	0.083 (–0.016, 0.183)	0.1265	2.686	0.106	0.39 (−0.07, 0.86)
Male size	1	–0.070 (–0.155, 0.015)	0.0002	0.003	0.954	–0.01 (–0.47, 0.45)
Iridescence	1	0.562 (–0.169, 1.293)	0.1105	2.346	0.130	0.36 (–0.10, 0.86)
Treatment×Male size	1	0.132 (0.018, 0.245)	0.2514	5.336	**0.024**	0.55 (0.08, 1.01)
Residual	72		3.3915			
***(c) Sigmoids***				*t*		
Treatment	1	0.245 (–0.706, 1.222)	–	0.508	0.613	0.12 (–0.34, 0.58)
***(d) Gonopodial thrusts***				*t*		
Treatment	1	–0.504 (–1.169, 0.122)	–	–1.544	0.127	–0.36 (–0.82, 0.17)
***(e) Copulation success rate***				*z*		
Treatment	1	0.605 (–0.486, 1.774)	–	1.068	0.286	0.25 (–0.21, 0.71)
Male size	1	0.932 (–0.102, 2.197)	–	1.632	0.103	0.38 (–0.09, 0.84)
Treatment×Male size	1	–1.414 (–2.920, –0.103)	–	−1.997	**0.046**	–0.47 (–0.93, 0.00)

Parameter estimates and effect sizes [59] are presented with 95% confidence intervals. Parameter estimates are relative to the high female availability (HFA) treatment level.

We also found no main effect of treatment on male sexual interest, but as with choosiness, our analysis revealed a significant interaction between treatment and male size on this behaviour (Table 2b). The Johnson-Neyman procedure indicated that only larger males responded to the female availability treatment, with LFA males showing the greatest sexual interest (Fig. 1b).

The number of sigmoid displays did not differ significantly between treatments (Table 2c), although the difference was in the expected direction, with LFA males tending to perform more sigmoids than HFA males. Similarly, the number of gonopodial thrusts did not differ significantly between treatments, although HFA males tended to perform more gonopodial thrusts than LFA males (Table 2d).

Despite low statistical power due to the low rate (25%) of mating success, we detected a marginally significant interaction between treatment and male size on copulation success, in which smaller LFA males and larger HFA males were more likely to successfully copulate during the 120 min mating trial (Table 2e).

### Postcopulatory Traits

Treatment had no significant effect on sperm counts, as estimated from the number of sperm stripped from the male (Table 3). Instead, copulation success was the only significant factor explaining variation in sperm counts in the final model (as expected, successful males had fewer remaining sperm). Similarly, there was no significant treatment effect on any measure of sperm quality (Table 4).

**Table 3:** Effect of female availability treatment and prior copulation success on the number of sperm in the guppy.

Source	*df*	Estimate	SS	*F*	*P*	Effect size
***Sperm count***						
Treatment	1	0.019 (–0.099, 0.137)	0.0065	0.104	0.748	0.08 (–0.40, 0.55)
Copulation success	1	–0.140 (–0.274, –0.005)	0.2695	4.298	**0.042**	–0.50 (–0.98, –0.02)
Residuals	70		4.3894			

Parameter estimates and effect sizes [59] are presented with 95% confidence intervals. Parameter estimates are relative to the high female availability (HFA) treatment level.

**Table 4:** Effect of female availability treatment on sperm quality traits in the guppy.

	Multivariate			Univariate						
Source	*Df*	Pillai	*P*	Sperm trait	Estimate	*df*	SS	*F*	*P*	Effect size
Treatment	1, 5, 59	0.051	0.680	Viability	–0.015 (–0.110, 0.080)	1	0.0001	0.003	0.960	–0.08 (–0.58, 0.42)
				Velocity	4.479 (–2.631, 11.59)	1	321.5	1.546	0.218	0.32 (–0.18, 0.82)
				Sperm head	0.017 (–0.029, 0.062)	1	0.005	0.641	0.427	0.18 (–0.31, 0.68)
				Sperm midpiece	–0.176 (–0.642, 0.291)	1	0.273	0.304	0.583	–0.19 (–0.69, 0.31)
				Sperm flagellum	0.367 (–0.243, 0.977)	1	1.504	0.983	0.325	0.30 (–0.20, 0.80)
Male size	1, 5, 59	0.184	**0.031**	Viability	0.066 (0.010, 0.122)	1	0.294	7.886	**0.007**	0.59 (0.09, 1.10)
				Velocity	1.661 (–2.540, 5.862)	1	94.20	0.453	0.503	0.20 (–0.30, 0.70)
				Sperm head	0.006 (–0.021, 0.033)	1	0.002	0.267	0.607	0.11 (–0.39, 0.61)
				Sperm midpiece	0.164 (–0.112, 0.440)	1	1.889	2.107	0.152	0.30 (–0.20, 0.80)
				Sperm flagellum	0.051 (–0.309, 0.412)	1	0.029	0.019	0.891	0.07 (–0.42, 0.57)
Iridescence	1, 5, 59	0.103	0.254	Viability	0.693 (–0.014, 1.401)	1	0.143	3.836	0.055	0.50 (–0.01, 1.00)
				Velocity	–17.015 (–69.84, 35.81)	1	86.10	0.414	0.522	–0.16 (–0.66, 0.33)
				Sperm head	0.057 (–0.280, 0.393)	1	0.0001	0.113	0.738	0.09 (–0.41, 0.58)
				Sperm midpiece	1.976 (–1.493, 5.445)	1	1.162	1.296	0.259	0.29 (–0.21, 0.79)
				Sperm flagellum	–4.674 (–9.204, –0.144)	1	6.500	4.251	**0.043**	–0.52 (–1.03, –0.02)
Residual				Viability		63	2.348			
				Velocity		63	1310			
				Sperm head		63	0.531			
				Sperm midpiece		63	56.48			
				Sperm flagellum		63	96.33			

Multivariate and univariate results are presented. Parameter estimates and effect sizes [59] are presented with 95% confidence intervals. Parameter estimates are relative to the high female availability (HFA) treatment level.

## Discussion

This study investigates how variation in the perceived level of future mating opportunities influences patterns of male reproductive investment in guppies, focusing on both pre- and postcopulatory traits. Contrary to expectations, we found no effect at the postcopulatory level (sperm number and quality), while our analyses of precopulatory traits revealed interacting effects of male body size and treatment on a number of traits (see Table 2). Broadly, large LFA males were more choosy, spent more time pursuing females, and had a lower likelihood of obtaining copulations relative to large HFA males. Our measure of choosiness also revealed that more coloured (iridescent) males, regardless of treatment, were more choosy than less colourful males.

Our finding that male size and female availability interact to determine precopulatory investment by males may be attributable to differences in male attractiveness. Larger males are usually preferred by female guppies [51] and other poeciliids [52]. Accordingly, larger (attractive) males may have enhanced mating success, and therefore be more sperm or energy limited compared to smaller (less attractive) males. If so, larger males may gain greater reproductive rewards by being choosy and tailoring their reproductive investment according to local mate availability [8]. Furthermore, the presence of precopulatory female choice may influence perceived female availability because less attractive males are more likely to be rejected by females [8]. Thus, all else being equal, relatively unattractive males may perceive lower mate availability than their more attractive counterparts. Our finding that more coloured (iridescent) males were on average more choosy, irrespective of treatment, is consistent with the idea that relatively attractive males are more discriminating. Attractiveness has already been shown to shape differential reproductive strategies in male guppies, as found in a recent study where the strength of male mate choice was related to a male’s own attractiveness [28]. It is worth noting that under our experimental conditions, our model selection did not select orange coloration, despite this being an important component of male attractiveness [13]. Nevertheless, female choice for male coloration varies extensively in this and other populations (e.g. [53], [54]) and the differences between our results and those reported by others might reflect these differences.

With lower expectations of future matings, males in the low female availability (LFA) treatment (or at least the larger ones) were more interested in females and focused more on the preferred female than their HFA counterparts. While it is intuitive that males in the LFA group should exhibit heightened sexual interest in females, it is less clear why they focused more on the preferred female. Theory predicts that males with higher perceived mating opportunities should be more discriminating, and thus exhibit enhanced choosiness in favour of the preferred female (reviewed by [6], [8], [9]). Our finding that males with fewer perceived future mating opportunities were more choosy suggests that instead of attempting to mate with as many females as possible, LFA males may focus on one female to maximise their chances of a successful mating. It is also worth noting that our data contained a strong positive correlation between choosiness and sexual interest (*R = *0.37, *P*<0.001) across both treatment groups, whereby males that exhibited more interest towards females also tended to focus more on the preferred female. It may be that the males that spent more time pursuing females have more time to judge the quality and the responsiveness of the two females, and as a consequence, are more likely to show marked preference. Clearly, this aspect of our findings deserves further investigation.

Despite the evidence that in the guppy, ejaculate traits are important in predicting male reproductive success [33] and can exhibit plasticity ([31]; but see [43]) we found no evidence for ejaculate tailoring in this study. There are at least three explanations for this. First, the absence of a postcopulatory response here may reflect the fact that males used in our experiment were all in good condition and fed ad libitum. There is evidence that males invest more prudently when their sperm reserves are depleted or their physical condition is poor (e.g. [55], [56]), including in the guppy, where courtship and sneaking rates are positively correlated with the size of sperm reserves [57], and males produce fewer sperm and of lower quality when food intake is experimentally limited [18], [19]. Future work that tests for adjustments in reproductive investment may benefit by subjecting males to dietary restriction. Second, the ability to adjust ejaculate traits may be limited to a shorter time frame than that imposed in the current experiment. Evidence from previous studies indicate that males are able to adjust both sperm production and velocity over a few days [30], [31] and therefore our longer treatment, which deliberately encompassed an entire spermatogenic cycle, may have masked such short-term adjustments. However, such plasticity was not found under a sperm competition scenario in a shorter period in the same population used in this study [43]. Third, variation in female availability may not be sufficient to trigger a plastic response in this species (for example, if natural fluctuations in female availability are not sufficient to drive the evolution of adaptive plasticity in males), with male reproductive strategies perhaps more strongly influenced by other factors predicting the reproductive return or the chances for a successful outcome of a mating, such as female mating status, fecundity, and social context [25], [27], [28], [58].

In conclusion, our findings reveal that tailoring of precopulatory male reproductive investment is more complex than predicted, with male strategies dependent on the interacting effects of male body size and the perceived level of future mating opportunities. By contrast, we found that sperm number and quality were unaffected by treatment. Taken together, these findings suggest that precopulatory rather than postcopulatory traits are most likely to be influenced by the perceived local availability of females.
